# Level of Awareness Regarding Interventional Radiology Among Medical Students at Northern Border University in Arar, Saudi Arabia

**DOI:** 10.7759/cureus.58512

**Published:** 2024-04-18

**Authors:** Pakeeza Shafiq, Yasir Mehmood, Ryanh H Alanazi, Rayan H Alanazi, Saja Alanazi, Rouh Maskhur K Alanazi

**Affiliations:** 1 Department of Surgery, Northern Border University, Arar, SAU; 2 College of Medicine, Northern Border University, Arar, SAU

**Keywords:** interventional radiology, awareness, medical students, arar, northern border university

## Abstract

Introduction

Interventional radiology (IR) is a highly specialized field of radiology that employs advanced imaging techniques like MRIs, CT scans, X-rays, and ultrasounds to detect and treat a variety of medical disorders. By using minimally invasive procedures, interventional radiologists can access the body's internal organs and tissues with minimal discomfort and reduced risks compared to traditional surgical techniques. Some common IR procedures include angioplasty, embolization, biopsy, and stent placement, among others. Overall, IR is an innovative and effective approach to medical care that offers numerous benefits to patients. As this specialty expands, there is a huge demand for increasing staff. However, due to a lack of awareness, this increased demand could not be fulfilled.

Objective

The objective is to assess medical students' knowledge regarding IR and compare this knowledge between male and female students.

Materials and methods

This cross-sectional study was carried out at Northern Border University's College of Medicine in Arar, Saudi Arabia. The study aimed to assess the medical students' knowledge of IR. All students enrolled in the clinical years at Northern Border University were included in the study, and a self-administered online questionnaire was used to collect data. The minimum sample size required was 169. Appropriate statistical analysis was applied to the collected data, and a p-value of less than 0.05 was considered significant.

Results

One hundred and seventy-two participants in all who met the inclusion criteria answered the study's questionnaire. The fourth-year students represented the highest percentage of the sample, with 65 participants (37.8%), followed by 54 (31.4%) fifth-year students and 53 (30.8%) sixth-year students. The study found that 66 participants (38.4%) rated their knowledge of IR as adequate, while only 8 (4.7%) considered it excellent. The participants' self-rated knowledge of IR did not significantly differ across male and female groups.

Conclusion

The study's findings suggest that medical students have limited knowledge of IR and that there is no discernible difference in the knowledge and interest of males and females in this subject. Further research and targeted educational interventions may be necessary to improve the medical students' overall knowledge and interest in IR.

## Introduction

Interventional radiology (IR) is a branch of radiology that uses various imaging techniques to diagnose and treat ailments with minimally invasive procedures. This field of medicine now encompasses a wide range of techniques. It includes placing central venous catheters, embolization of arteries and veins for controlling hemorrhage, image-guided biopsy, image-guided percutaneous drainage, image-guided drain placement, and image-guided ablation and chemotherapy of tumors [1,2]. These procedures conducted by interventional radiologists replace the major surgical procedures. The interventional radiologist reaches the hard target areas by using needles, catheters, and wires. The advantages of these procedures are shorter stay in the hospital, early return to work, and lesser risks of open surgery. Angioplasty, embolization procedures, drainage, ablation treatments, and stent insertion are all done by IR. However, the overlapping of IR and other fields for these procedures has mainly led to unclear perceptions of medical students [3]. 

The American Board of Medical Specialties recognized IR as a distinct specialty in 2012, and a residency program was established. Despite its ever-expanding umbrella, physicians were either not informed or misinformed. As a result, further down the ladder, the medical students remained uninformed [4,5]. The slow or non-propagation of IR among medical professionals themselves has led to a scant population of interventional radiologists. Hence, this small group has to face a huge burden of patients at their disposal. This has also been reported in the United Kingdom workforce report of 2016. It was also noted that the level of enrollment in universities for this clinical field was also low owing to a lack of familiarity with this field [6]. 

The increasing use of IR warrants the teaching of its basics to undergraduate students. On the contrary, the medical education is limited to diagnostic radiology. Various previous studies warrant further initiative in adding to the IR curriculum. A Canadian study showed that 91% of students requested an addition to the IR syllabus [7]. According to a study conducted in England, there is a significant knowledge gap among students regarding IR when compared to other specialties. Only 10.9% considered IR as a career [8]. This highlights an opportunity for educators to focus on improving the understanding and awareness of this important field. A recent study in Saudi Arabia showed that 61% of students favored increasing exposure to IR [9]. In addition to the uncertainty surrounding the area, the medical literature contains limited studies on medical students' awareness of IR, especially those from the Middle East [10].

This study aims to assess medical students' knowledge regarding IR and compare this knowledge between male and female students. This study will add to national data already present and help in formulating guidelines or improving the curriculum of undergraduate medical students regarding this specific field of medicine.

## Materials and methods

Study design and setting

This is a cross-sectional study that was conducted at Northern Border University's College of Medicine in Arar, Saudi Arabia, after obtaining approval from its Local Committee of Bioethics (approval number: 58/44/H). This study was completed over a period of six months from July 2023 to December 2023. The study aimed to assess the knowledge of medical students about IR.

Inclusion and exclusion criteria

The medical students of Northern Border University in their clinical years, i.e., fourth, fifth, and sixth years, were included in the study, while students in junior years were excluded. The students' consent was taken alongside the questionnaire as complete submission meant consent to be included in the study. Those who did not give consent or did not complete the questionnaire were excluded from the study. They were informed that data would be confidential.

Sampling technique and sample size

Medical students were selected using a non-probability convenient sampling technique. The calculated sample size was 169 with 95% CI and 5% margin of error. The survey received 172 responses.

Data collection tool

The tool used to collect data was a self-administered online questionnaire (see Appendices). This questionnaire was adapted from a previous study conducted in Canada by O'Malley and Athreya [11]. There were 17 questions in the survey, divided into two parts: demographics and IR questions. Demographic data, including gender and academic year, were covered in the first part. In the second part, students were assessed on their knowledge of IR and training. The survey aimed to assess their level of knowledge about IR, their interest in it, and their willingness to learn more about this subject. The respondents were asked to share how they had learned about IR and to indicate their preferred learning strategies. Furthermore, the survey aimed to gauge the respondents' interest in participating in a possible elective rotation in IR. The questionnaire was then converted to an online form using Google Forms and distributed to students through WhatsApp.

Statistical analysis

IBM SPSS Statistics for Windows, Version 24.0 (Released 2016; IBM Corp., Armonk, New York, United States) was used to enter and analyze the data. We used the Shapiro-Wilk test to determine the normality of data. We presented qualitative variables in the form of frequency and percentages. We used the chi-squared analysis to compare the proportions of qualitative variables between the two groups. A p-value less than 0.05 was considered significant. 

## Results

Table 1 shows that out of 172 respondents who participated in the study, 87 (50.6%) were females, while 85 (49.4%) were males. Regarding the study year, there were three groups: 65 (37.8%) in their fourth year, 54 (31.4%) in their fifth year, and 53 (30.8%) in their sixth year. Regarding whether they have done or are aiming to do an elective in radiology, 67 participants (39%) answered yes, while 105 (61%) answered no. As far as their interest in diagnostic radiology as a career is concerned, 33 participants (19.2%) answered yes, 66 (38.4%) answered no, and 73 (42.4%) were not sure. Among those who answered no or not sure, the most common reasons given were not finding it interesting (55 participants, 32%) and not knowing enough about it (64 participants, 37.2%). Other reasons included not liking the lifestyle (28 participants, 16.3%) and concerns about radiation exposure (25 participants, 14.5%). Finally, 86 participants (50%) showed interest in doing an elective rotation in IR, 30 (17.4%) didn't show interest, and 56 (32.6%) were not sure.

**Table 1:** General characteristics of the participants (n=172)

Characteristics	Frequency and proportions n (%)
Gender	
Male	85 (49.4%)
Female	87 (50.6%)
Year of the study	
Fourth	65 (37.8%)
Fifth	54 (31.4%)
Sixth	53 (30.8%)
Plan to do an elective in interventional radiology	
Yes	67 (39%)
No	105 (61%)
Consider diagnostic radiology as a career option	
Yes	33 (19.2%)
No	66 (38.4%)
Not sure	73 (42.4%)
Reason for not choosing radiology as a career	
It is not interesting	55 (32%)
Insufficient knowledge	64 (37.2%)
Unsuitable lifestyle	28 (16.3%)
Radiation exposure	25 (14.5%)
Interest in doing a two-week interventional radiology elective	
Yes	86 (50.0%)
No	30 (17.4%)
Not sure	56 (32.6%)

Table 2 shows that 66 participants (38.4%) rated their knowledge of IR as adequate, while only eight (4.7%) considered it excellent. Thirty-six participants (20.9%) rated their knowledge as good, 17 participants (9.9%) reported having no knowledge, and 45 (26.2%) rated their knowledge as poor. Regarding exposure to patients treated by interventional radiologists, 58 participants (33.7%) answered "yes," while 83 (48.3%) answered "no," and 31 (18%) were not sure. In addition, 52 participants (30.2%) disagreed with the statement made by 120 participants (69.8%) about interventional radiologists' outpatient clinics. Similarly, 85 respondents (49.4%) disagreed with the statement made by 87 respondents (50.6%) that ward rounds are essential for interventional radiologists. In terms of interventional radiologists admitting patients, 110 participants (64%) agreed with the statement, while 62 (36%) did not agree. On the other hand, 110 participants (64%) said that a two-week radiology rotation would be helpful, 39 (22.7%) were not sure, and 23 (13.4%) indicated that it would not be beneficial.

**Table 2:** Proportion of interventional radiology level of awareness among the participants (n=172)

Characteristics	Frequency and proportions n (%)
Rate your knowledge about interventional radiology	
Adequate	66 (38.4%)
Excellent	8 (4.7%)
Good	36 (20.9%)
No knowledge	17 (9.9%)
Poor	45 (26.2%)
Have you seen patients who were treated by an interventional radiologist?	
Yes	58 (33.7%)
No	83 (48.3%)
Not sure	31 (18.0%)
Required residency for interventional radiologist	
Radiology	68 (39.5%)
Surgery	11 (6.4%)
Both radiology and surgery	76 (44.2%)
Internal medicine	15 (8.7%)
Dermatology	2 (1.2%)
Outpatient clinics in interventional radiology	
Yes	120 (69.8%)
No	52 (30.2%)
Ward rounds in interventional radiology	
Yes	87 (50.6%)
No	85 (49.4%)
Patients' admission by interventional radiologists	
Yes	110 (64%)
No	62 (36%)
Two-week radiology rotation is beneficial	
Yes	110 (64%)
No	23 (13.4%)
Not sure	39 (22.7%)

Table 3 shows that 33 participants (19.2%) reported that the most common source of information about IR was lectures from interventional radiologists and self-directed research. Other sources reported were radiology elective by 24 respondents (14%), ward rounds in the hospital by 21 respondents (12.2%), problem-based learning by 13 respondents (7.6%), and multidisciplinary meetings by four participants(2.2%). Finally, 44 participants (25.6%) reported having no exposure to IR.

**Table 3:** Source of information about interventional radiology (n=172)

Source of information regarding interventional radiology	Frequency and proportions n (%)
Radiology elective	24 (14%)
Interventional radiology lectures	33 (19.2%)
Problem-based learning	13 (7.6%)
Research	33 (19.2%)
Hospital ward rounds	21 (12.2%)
Multidisciplinary meetings	4 (2.2%)
No information about interventional radiology	44 (25.6%)

Table 4 shows that the most favored method was ward rounds, with 98 participants (57%) ranking it as their top choice. Next was the radiology department, with 85 participants (49.4%) ranking it as their favorite method. Electives were also popular, with 59 participants (34.3%) ranking it as their top choice. Lectures and multidisciplinary meetings were also highly ranked by the participants. Research and problem-based learning were less favored, with only 32 participants (18.6%) and 29 participants (16.9%) ranking them as their top choice, respectively. Tutorials and clinical research projects were popular among 45 participants (26.2%) and 47 participants (27.3%), respectively.

**Table 4:** Methods considered favorite to learn about interventional radiology by the participants (n=172) (from 1 (best) to 8 (worst))

Methods	1	2	3	4	5	6	7	8
Ward rounds	98 (57%)	23 (13.4%)	27 (15.7%)	12 (7.0%)	4 (2.3%)	1 (0.6%)	2 (1.2%)	8 (2.9%)
Radiology department	85 (49.4%)	37 (21.5%)	31 (18.0%)	9 (5.2%)	2 (1.2%)	3 (1.7%)	2 (1.2%)	3 (1.7%)
Electives	59 (34.3%)	35 (20.3%)	37 (21.5%)	18 (10.5%)	11 (6.4%)	5 (2.9%)	1 (0.6%)	6 (3.5%)
Lectures from interventional radiologists	48 (27.9%)	26 (15.1%)	47 (27.3%)	24 (14%)	9 (5.2%)	6 (3.5%)	6 (3.5%)	6 (3.5%)
Multidisciplinary meetings	38 (22.1%)	33 (19.2%)	50 (29.1%)	25 (14.5%)	9 (5.2%)	9 (5.2%)	3 (1.7%)	5 (2.9%)
Self-directed learning websites	32 (18.6%)	26 (15.1%)	41 (23.8%)	19 (11%)	10 (5.8%)	12 (7.0%)	14 (8.1%)	18(10.5%)
Problem-based learning	29 (16.9%)	28 (16.3%)	41 (23.8%)	21 (12.2%)	9 (5.2%)	11 (6.4%)	13 (7.6%)	20(11.6%)
Tutorials	45 (26.2%)	45 (26.2%)	40 (23.3%)	16 (9.3%)	9 (5.2%)	4 (2.3%)	9 (5.2%)	4 (2.3%)
Clinical research projects	47 (27.3%)	27 (15.3%)	48 (27.9%)	23 (13.8%)	9 (5.2%)	6 (3.5%)	6 (3.5%)	6 (3.5%)

Table 5 shows that regarding the self-rated knowledge of IR between males and females, there was no statistically significant difference (p=0.921). The percentage of males and females who had seen patients treated by an interventional radiologist also did not differ significantly (p=0.438). There were no differences between male and female participants' awareness of specific characteristics of IR (e.g., whether these professionals conduct hospital ward rounds or have outpatient clinics) (p=0.762 and p=0.119, respectively). There was no significant difference when it came to whether interventional radiologists actually admit patients to hospitals (p=0.248). There were no discernible differences between the genders in terms of the sources of information they used to learn about IR (p=0.654). Both genders most frequently cited self-directed research and lectures as sources of information. Furthermore, when asked if a mandated two-week radiology rotation during clerkship would be advantageous, it was not significant (p=0.866). The percentage of males and females who would like to learn more about IR also did not significantly differ (p=0.496).

**Table 5:** Relationship between gender and awareness of interventional radiology (n=172) *: p-value <0.05 (statistically significant)

Characteristics	Male n (%)	Female n (%)	P-value*
Rate your knowledge about interventional radiology			0.921
Adequate	35 (53.0%)	31 (47.0%)	
Excellent	4 (50%)	4 (50%)	
Good	20 (55.6%)	16 (44.4%)	
No knowledge	8 (47.1%)	9 (52.9%)	
Poor	24 (53.3%)	21 (46.7%)	
Have you seen patients who were treated by an interventional radiologist?			0.438
Yes	27 (46.6%)	31 (53.4%)	
No	45 (54.2%)	38 (45.8%)	
Not sure	13 (41.9%)	18 (58.1%)	
Outpatient clinics in interventional radiology			0.119
True	64 (53.3%)	56 (46.7%)	
False	21 (40.4%)	31 (59.6%)	
Ward rounds in interventional radiology			0.762
True	45 (51.7%)	42 (48.3%)	
False	43 (50.6%)	42 (49.4%)	
Patients' admission by interventional radiologists			0.248
True	58 (52.7%)	52 (47.3%)	
False	27 (43.5%)	35 (56.5%)	
Source of information regarding interventional radiology			0.654
Radiology elective	9 (37.5%)	15 (62.5%)	
Interventional radiology lectures	19 (57.6%)	14 (42.4%)	
Problem-based learning	5 (38.5%)	8 (61.5%)	
Research	15 (45.5%)	18 (54.5%)	
Hospital ward round	12 (57.1%)	9 (42.9%)	
Multidisciplinary meetings	1 (33.3%)	2 (66.7%)	
No information about interventional radiology	23 (52.3%)	21 (47.7.9%)	
Two-week radiology rotation is beneficial			0.866
Yes	56 (50.9%)	54 (49.1%)	
No	18 (46.2%)	21 (53.8%)	
Not sure	11 (47.8%)	12 (52.2%)	

## Discussion

The students' knowledge and awareness regarding this specialty have been evaluated extensively both internationally and locally. All of the studies confirmed that medical students lack a good understanding of it. Arar City, however, has not conducted any similar studies. This study shows that 38.4% of the participants rated their knowledge of IR as adequate, while only 4.7% considered it excellent. The results are better than those reported in other studies, which indicated that a greater percentage of knowledge was poor or nonexistent, including O'Malley and Athreya and Albaqawi et al. who found that 55% of participants and 45% of respondents had poor knowledge and information about IR, respectively [11,12]. According to Atiiga et al.'s findings, 55.5% of students thought they didn't know enough about IR [13]. Thirty-nine percent of our survey respondents had completed or planned to complete an elective in radiology, and 19.2% considered a career in radiology. The lack of knowledge of IR (37.2%), loss of interest (32%), and concerns about radiation (14.50%) were the primary reasons for not considering a career in IR. The study by O'Malley and Athreya found that 59% of students had completed an elective in radiology or were planning to do so. The respondents were 18% interested in IR careers and 22% in radiology careers. Almost half of them (48%) cited lacking knowledge as a major reason for not considering a career in IR, and nearly four in 10 (43%) cited lack of interest [11]. There was only 17% interest in diagnostic radiology or IR (13.5%) in Albaqawi et al. survey results; however, most of the participants were not interested in IR; lack of information about the specialty was the most common reason (27.5%) for respondents not being interested [12]. According to the European surveys, a respectable portion of participants (41%) are open to the idea of pursuing a career in IR [14]. However, just 13% of medical students in the United States expressed interest in pursuing a career in IR [15].

Sixty-nine percent of medical students in Nigeria were found to know very little about IR. As a result, this ignorance contributed to a lack of interest in IR as a career [16]. The medical students in India demonstrate a similar level of lack of awareness of IR: about 60%. This is mostly linked to a lack of teaching to undergraduate students [17].

A comprehensive review of research evaluating medical students' knowledge of IR revealed that more than half of them rated their understanding as inadequate. Research conducted following tutorial courses revealed a considerable improvement in the students' interest and knowledge [18]. As of right now, medical schools (including those in China and the United States) do not have any established, appropriate organized teaching modules in their curricula [19]. A lack of knowledge of IR among undergraduate students could explain the low workforce in the IR departments [20]. There are several approaches to address the issue of medical students' inadequate understanding of IR, including raising awareness, exposing students to the field, and stimulating their interest in it from the very beginning. In the United States, there was a strong response to the idea that radiology courses should be included in the curriculum [21]. Educating radiology has a positive effect on medical students' interest in, perception of, and attitude towards IR, even during the preclinical years of their education [22,23].

The study's small sample size and exclusive focal institution limited the findings' applicability to a wider range of medical students. It might be essential to collect a larger sample size with the inclusion of other institutions across the country in order to get precise and broadly applicable results.

Participants may have provided responses based on social desirability or perceived expectations rather than their true knowledge and interest in IR, resulting in self-reporting bias. These should be taken into consideration in future research to solve these potential shortcomings.

## Conclusions

The study's findings suggest that medical students have limited knowledge of IR and that there is no discernible difference in the knowledge and interest of males and females in this subject. Further research and targeted educational interventions may be necessary to improve the medical students' overall knowledge and interest in IR.

The strategies to improve medical students' understanding of and interest in IR include integrated curriculum, elective rotations, guest lectures and workshops, mentorship programs, and provision of educational resources such as seminars and literature to develop students' understanding of IR. By implementing these strategies, medical schools can help improve the understanding and interest of their students in IR.

## Appendices

Date: __________________

Age: ___________________

Gender: male/female

1. Year of medical school: 4/5/6

2. How would you rate your knowledge of interventional radiology as compared to other subjects?

Excellent/good/adequate/poor/no knowledge

3. Have you completed or do you plan to complete an elective in radiology (diagnostic or interventional)?

Yes

No

4. Would you consider a career in diagnostic radiology?

Yes

No

Not sure

5. Would you consider a career in interventional radiology?

Yes

No

Not sure

If you answered no or not sure to the previous question, please choose the most appropriate reason why.

I don't find it interesting

I don't know enough about it

The lifestyle is not for me

Radiation exposure

7. Have you seen patients who were treated by an interventional radiologist?

Yes

No

Not sure

8. Please list three interventional radiology procedures that you are aware of:

1. ____________________

2. ____________________

3. ____________________

9. An interventional radiologist must complete a residency in:

Radiology

Surgery

Both radiology and surgery

Internal medicine

Others (please specify): _____________________

10. Interventional radiologists have outpatient clinics.

True

False

11. Interventional radiologists do ward rounds in the hospital.

True

False

12. Interventional radiologists admit patients to the hospital.

True

False

13. What has provided you with the most information about interventional radiology?

Radiology elective

Lectures from interventional radiologists

Problem-based learning tutorials

Self-directed research

Ward rounds in the hospital

Multidisciplinary meetings

I have had no exposure to interventional radiology.

Others (please specify): ______________________

14. How would you prefer to gain exposure to interventional radiology? Please rank the following methods for learning

(rank 1 (best) to 8 (worst)).

1 (best)/2/3/4/5/6/7/8 (worst)

Ward rounds

Radiology department

Electives

Lectures from interventional radiologists

Multidisciplinary meetings

Self-directed learning websites

Problem-based learning

Tutorials

Clinical research projects

15. Do you think a mandatory two-week radiology rotation during clerkship would be beneficial?

Yes

No

Not sure
